# Supplementary material for: The effects of risk-taking, exploitation, and exploration on creativity
Source: PLoS One. 2020 Jul 30;15(7):e0235698. doi: 10.1371/journal.pone.0235698 (PMC7392310; doi:10.1371/journal.pone.0235698)
Supplement: Attachment — Submitted filename: review_PONE-D-20-02635_Skvortsova.docx [file pone.0235698.s001.docx]

The manuscript addresses the question of how two measures of creativity (divergent vs. convergent) thinking relates to exploration – exploitation strategies in the version of Iowa Gambling Task. The author made a prediction that divergent thinking measured through the S-A creativity test is related to more exploratory behavior while convergent thinking should bias behavioral strategies towards more exploitation. However, the obtained results do not confirm this hypothesis. Individuals who scored more on divergent thinking show some relationship to both exploration and exploitation (which depends on the chosen Q-learning model) while convergent thinking does not show any relationship with exploration-exploitation trade-off in this particular task.

The author then made some post-hoc interpretations trying to reconcile the obtained results. Despite the fact that I appreciate the effort of trying to investigate the link between such a complex phenomenon as creativity and learning, these results alone do not lead to any conclusion and do not provide any clear guidelines to future research and, hence, could not be published.

Some methodological points:

- No details about model-fitting procedures and model selection are provided. The reader does not know whether any of two proposed Q-learning models better explained subject’s behavior and how did it account for the data in general. In this context analysis of exploitation-exploration trade-off becomes difficult since labeling of choices is based on Q-values and poorly fitted model would predict more exploratory decisions.
  There is no information about the values of learning rates and other parameters and no analysis of subjects’ behavior: how well they performed on the task.
- All Q-values for all four bandits are initiated at 0 which might bias Q-value estimates. The bandits have different expected values and different variances which makes the interpretation of prediction errors quite difficult (e.g. more variable bandits generate larger prediction errors). If the author decided to use this particular task, the choice should be justified.
- There is no information whether the predictors were z-scored before entering the regression analysis which makes the interpretation of regression coefficients problematic.
