# Supplementary material for: The effects of risk-taking, exploitation, and exploration on creativity
Source: PLoS One. 2020 Jul 30;15(7):e0235698. doi: 10.1371/journal.pone.0235698 (PMC7392310; doi:10.1371/journal.pone.0235698)
Supplement: Attachment — Submitted filename: Revision list.docx [file pone.0235698.s002.docx]

**Revision List**

We greatly appreciate reviewers’ helpful comments and we believe we could significantly improve the quality of the paper. Here are how we revised the manuscript in response to the comments.

Main changes
First, the main revisions in the current manuscript are as follows:

1. In response to Reviewer 2’s comment on the potential influence of risk taking on creativity, we reformulated the Q learning model incorporating the simplified version of the prospective utility function, in which risk attitudes are measured parametrically, and examined their effects on creativity. As a result, we completely deleted the simple Q learning model in the previous paper and the time-varying model. Instead, we only considered this new model alone in order to examine the effects of risk taking on creativity.
2. In response to Reviewer 3’s suggestion, we reexamined the learning convergence for each participant, and once the convergence was identified, we dropped the subsequent data in the IGT. Then, we split the sample into two subsamples: the sample of learning convergence and that of non-convergence, and examined the determinants of creativity in each subsample.
3. In the estimation, following the comment by Reviewer 3, we added inverse temperature to substitute for exploitation, exploration, and exploration/exploitation (which are also questioned by Reviewer 2). We only examined the effect of the inverse temperature that is considered to reflect exploitation/exploration ratio in the related studies.
4. Following Reviewer 2’s comments, we added most of the literature suggested by Reviewer 2 in reviewing the related studies.
5. As a result, the current manuscript examined the effects of not only exploitation/exploration but also risk-taking on creativity. Hence, we changed the title of the paper as “The effects of risk-taking, exploitation, and exploration on creativity

Responses to Reviewer 1:

Reviewer 1 raised the question as “Despite the fact that I appreciate the effort of trying to investigate the link between such a complex phenomenon as creativity and learning, these results alone do not lead to any conclusion and do not provide any clear guidelines to future research and, hence, could not be published.”

- In the current version, the main research results shifted from exploitation/exploration to risk taking where a number of related studies examined. Our results were consistent with one of the related studies, and not consistent with the other. Given these results, we discussed why these differences arose. In addition, in contrast to the related studies, this manuscript adopted different alternative measures for risk-taking. We believe providing this alternative measures and empirical studies enrich existing literature on risk-taking and creativity.

Methodological comment #1: “No details about model-fitting procedures and model selection are provided….”

- In the current version, only the single model was considered so that the model comparison is not needed.

Methodological comment #2: “There is no information about the values of learning rates and other parameters and no analysis of subjects’ behavior: how well they performed on the task.”

- Estimation was made for all 113 participants and we summarized these data in Table 1 as means and SDs. Regarding the performance, we added learning convergence data in the current version.

Methodological comment #3: “All Q-values for all four bandits are initiated at 0 which might bias Q-value estimates. The bandits have different expected values and different variances which makes the interpretation of prediction errors quite difficult (e.g. more variable bandits generate larger prediction errors). If the author decided to use this particular task, the choice should be justified.”

- We believe the initial value of each deck should be zero because participants have no information regarding rewards and probabilities before starting the game. This assumption seems reasonable and also consistent with related studies giving starting Q values as zero. Even if we set the starting values at K (>0) for all participants, the statistical results should remain the same, though there might emerge some level effects. That is, the relative ranking of parameter estimates should remain the same as long as the same starting values are given to all participants.
- We used the IGT rather than the two armed bandit because in the latter case, participants easily achieved learning convergence during 100 trials based on our pre-experiments.

Methodological comment #4: “There is no information whether the predictors were z-scored before entering the regression analysis which makes the interpretation of regression coefficients problematic.”

- This regression analysis used the parameter estimates as they are, so we did not standardize these parameters. We believe if the magnitudes of the estimates matter in the analysis, the standardization such as Z scores might make sense. However, this study only examine the statistical relationship between risk-taking, inverse temperature and creativity, we think we do not necessarily have to resort to the standardization procedure.

Responses to Reviewer 2 (Prof. Hommel):

His comments (#1~#3) provided related literature that was missing in the previous manuscript.

- We cited most of the studies suggested by Reviewer 2 in the relevant places. We believe this significantly clarified the relation of the current paper to the existing literature.

Comment #4: “The parameter is used to indicate exploitation and exploration are likely to be affected, if not confounded with risk taking. Given that risk-taking has an impact on creativity, this is a serious problem:…”

- We took this comment quite seriously, and incorporated the risk components in our model by incorporating the simplified version of the prospective utility function. Then we examined their effects. As Reviewer 2 correctly anticipated, we could only confirm the effects of risk-taking, and those of inverse temperature disappeared once the risk-taking variables were added in the regression.

Comment #5: “It remains unclear what the ratio between optimal and non-optimal choices is supposed to represent theoretically/mechanistically.”

- We completely dropped exploitation/exploration ratio, and considered the inverse temperature instead.

Comment #6: “Divergent thinking was assessed by means of a non-established, self-developed task that doesn't seem to be validated. Why was AUT not used instead?”

- We are sorry that our description regarding the S-A test was not sufficiently clear. The first section of the S-A test is actually the AUT. So we made this clear by writing “In the first task, participants are instructed to generate alternative ways of using objects specified in the test, which is known as alternative use tests.” (p. 10).

Comment #7: “It remained unclear how the findings shown in tables 2 and 3 were generated. …. a) the predictors are obviously highly interrelated, at least in several cases, which is an absolute no go for regression analyses; b) this analysis can only assess linear effects, even though nonlinear functions seem to play a much more decisive role :…”

- First, in the current version, only a single model was considered.
- Second, correlation matrix was provided in Table 1, and though risk parameters and inverse temperature are highly correlated, the overall correlation did not seem to generate a singularity of the matrix of regressors.
- Third, following the work by Akbari Chermahini, S., & Hommel, B. (2010; 2012) suggested by Reviewer 2, we also included the squared exogenous variables to check the nonlinear relationship such as the inverted U shape reported in these papers.

Comment #8: “It remains unclear which outcome suggests that "divergent thinkers pursue both exploitation and exploration simultaneously". The fact that the ratio is involved does not speak to this issue at all, as the values are likely to represent different trials.”

- We dropped this sentence.

Comment #9: “The two models do not replicate each other. Given that they are theoretical status is unclear, this means that be don't know what the outcome means.”

- Now, only one model is considered.

Comment #10: “In the discussion, there are repeatedly Chinese characters mentioned. I had no idea where they come from. In the description of the RAT, Chinese characters were not mentioned, and I found it difficult to imagine an RAT in which Chinese characters play a role.”

- We were sorry that this was not clearly described because we though the Japanese version of the RAT automatically imply Chinese characters, which should not be presupposed. So, we change the sentence as follows in p. 11: “…we adopted a Japanese version of RAT developed by (39) where words were represented by Chinese characters.”

Responses to Reviewer 3:

Comment #1: “Do the subjects learn to pick the best card(s) eventually and if so when (at which trial) are they starting to select the best option(s) consistently? This aspect is critical, particularly as the task is quite long, if subjects learn which is the best option after 20 trials, it is very likely that the choices made in the 80 remaining trials will be defined as exploitation, and that would just be the results of subjects learning to play the task and not a strategy that could be opposed to exploration. Depending on this result, the author could consider doing a similar analysis focusing on the learning phase (before reaching the plateau) that is maybe occurring during the first 30/50 trials? This may not be necessary but showing the performance of subjects is the only way to make sure of that.”

- Following this suggestion, we examined the learning convergence and examined the determinants of creativity in the two subsamples. We also added Section 3.1. Learning convergence, to describe it.
- We reported there that the average number of trials before learning convergence was 70.9 (SD=29.5).

Comments #2 ~#4 were regarding the selection and comparison between the two models in the previous manuscript.

- In the current version, we only considered the one model with the prospective utility function, so though these comments were quite important and helpful, the current manuscript did not have to consider these issues.

Comments #5: “Exploration in the paper is rather extremely defined as the number of choices of the option with the lowest Q-value. That makes, for instance, a choice of the second worst option, not an exploratory one. Can the author justify this narrow definition of an explorative choice? “

- We dropped our previous measures of exploitation and exploration in the current version.

Comment #6: “In the reinforcement learning literature, the temperature (or inverse temperature here) can be interpreted as a parameter governing the exploration/exploitation tradeoff. “

- Following this advice, we replaced exploitation/exploration by the inverse temperature.

Minor concerns #1: “Page 6 of the manuscript, it is written that the average loss concerning the two disadvantageous decks is -$50, is it not -$25 instead?”

- We found some of the numbers reported here were wrong. We have corrected them.

Minor concerns #2: “Concerning the second model, the dynamic learning rates seem not to be bounded between 0 and 1, is it the case? If it is, I think it would be important to consider adding details about parameters bounds.

- It is correct that the learning parameters were bounded between 0 and 1, by imposing the beta distribution in the MAP estimation. In the current version, because the time-varying learning model was dropped, we did not describe the details about parameters bounds in this model.

Minor concerns #3: “Page 10, last sentence of the Asymmetric time-varying Q-Model paragraph, equations references seem to be wrong, they should be 4 and 5 instead of 6 and 5.”

- We dropped this part because this model was not considered in the current version.

Minor concerns #4: “Pages 18 and 22, the numbers of stars in the legend of the table are erroneous. One star for p<0.1 and for p<0.05”.

- This was corrected.
